# Statistical analysis plans for two randomised controlled trials of the Narrative Experiences Online (NEON) Intervention: impact of receiving recorded mental health recovery narratives on quality of life in people experiencing psychosis (NEON) and people experiencing non-psychosis mental health problems (NEON-O)

**DOI:** 10.1186/s13063-023-07246-8

**Published:** 2023-05-20

**Authors:** Clare Robinson, Chris Newby, Stefan Rennick-Egglestone, Joy Llewellyn-Beardsley, Fiona Ng, Rachel A. Elliott, Mike Slade

**Affiliations:** 1 Centre for Evaluation and Methods, Pragmatic Clinical Trials Unit, Queen Mary University of London, London, UK; 2 School of Medicine, University of Nottingham, Nottingham, UK; 3 Institute of Mental Health, School of Health Sciences, University of Nottingham, Nottingham, UK; 4 Manchester Centre for Health Economics, School of Health Sciences, University of Manchester, Manchester, UK; 5 Faculty of Nursing and Health Sciences, Health and Community Participation Division, Nord University, Namsos, Norway

**Keywords:** Recovery narrative, Psychosis, Mental health, Statistical analysis plan, Online intervention

## Abstract

**Background:**

Mental health recovery narratives are a first-hand account of an individual’s recovery from mental health distress, access to narratives can aid recovery. The NEON Intervention is a web-application providing access to a managed collection of narratives. We present the statistical analysis plan for assessing the effectiveness of the NEON Intervention in improving quality of life at 1-year post-randomisation. We pay particular focus on the statistical challenges encountered due to the online nature of this trial.

**Methods and design:**

The NEON Intervention is assessed in two trial populations, one for people with experience of psychosis in the last 5 years, and mental health distress in the last six months (NEON Trial) and one for people with experience of non-psychosis mental health problems (NEON-O Trial). Both NEON trials are two-arm randomised controlled superiority trials comparing the effectiveness of the NEON Intervention with usual care. The target sample size is 684 randomised participants for NEON and 994 for NEON-O. Participants were randomised centrally in a 1:1 ratio.

**Results:**

The primary outcome is the mean score of subjective items on the Manchester Short Assessment of Quality-of-Life questionnaire (MANSA) at 52 weeks. Secondary outcomes are scores from the Herth Hope Index, Mental Health Confidence Scale, Meaning of Life questionnaire, CORE-10 questionnaire and Euroqol 5-Dimension 5-Level (EQ-5D-5L).

**Conclusion:**

This manuscript is the statistical analysis plan (SAP) for the NEON trials. Any post hoc analysis, such as those requested by journal reviewers will be clearly labelled as such in the final trial reporting.

Trial registration

Both trials were prospectively registered. NEON Trial: ISRCTN11152837, registered on 13 August 2018. NEON-O Trial: ISRCTN63197153, registered on 9 January 2020.

## Introduction

Mental health recovery narratives are a first-hand account of an individual’s recovery from mental health distress. Narratives can be recorded in text, audio, or video form and collections of narratives are publicly available [1–5]. Recovery narratives can be shared in person or online to aid the recovery of others. Receiving a recovery narrative can provide personal inspiration [6], increase empathy and understanding [7], validate difficult personal experiences [8], or provide alternative forms of companionship [9]. They also may contribute to recipient distress if, for example, the recipient perceives themselves to have experienced greater hardship or shown less resilience than the narrator [6]. There has been no randomised controlled trial (RCT) to determine the benefits of recorded recovery narratives for people experiencing psychosis.

The Narrative Experiences Online (NEON) Intervention is a web-application providing access to a managed collection of recorded mental health recovery narratives, referred to as the NEON Collection [10]. It was developed for three groups: people with experience of psychosis, which is to be evaluated in the definitive NEON Trial; people with experience of non-psychosis mental health problems, to be evaluated in the definitive NEON-O Trial; and informal carers, to be evaluated in the NEON-C feasibility trial (reported elsewhere). The NEON Trial and NEON-O Trial are referred to collectively as the NEON trials.

The aim of the NEON trials is to investigate whether receiving recorded recovery narratives can improve quality of life at 1 year as measured by the Manchester Short Assessment of Quality of Life questionnaire (MANSA) [11], compared to usual care, for those with experience of psychosis in the last 5 years, and mental health distress in the last six months (NEON Trial) and for people with experience of non-psychosis mental health problems (NEON-O Trial).

The NEON and NEON-O Trials opened in March 2020 and the studies were completed in September 2022. Both trials were conducted completely online. Participants were recruited via a diverse range of methods such as online advertising, the distribution of digital messages through social media platforms, advertising in print media, placement of posters and leaflets in community venues, approach by clinical support officers, and recommendations by health care professionals. All recruitment activity resulted in a participant being directed to the web address of the splash page for the NEON trials. From here, a participant completed online eligibility and consent procedures, for which they had to provide a valid email address. If eligible for one of the NEON trials the participant was randomised via the web application, after completing all baseline data questionnaires. All further follow-up data was collected via online questionnaires.

Conducting a trial completely online offers many advantages in terms of reduced costs, automated data collection and the ability to include individuals across a large geographical area. However, it can introduce new challenges across aspects of the trial including recruitment, randomisation, intervention fidelity, retention, and data quality [12–14]. Of particular importance for statistical considerations is the potential for participants to register more than once for the trial and hence be randomised multiple times; participants to not engage with the intervention; low retention rates and the potential for differential follow-up across treatment arms; and poor accuracy of data due to being unable to independently validate or query any particular response.

Almost the entirety of the NEON trials was conducted during the COVID-19 global pandemic. Due to the online nature of the NEON trials the pandemic had little impact upon the administration or operational procedures of the NEON trials, although it did cause a change in the balance of recruitment work towards online recruitment.

Here we present the statistical analysis plan (SAP) for the clinical effectiveness of the NEON and NEON-O trials, with particular focus on some of the unique statistical challenges encountered due to the online nature of the trials.

This SAP was written following the guidelines for the statistical analysis plans by Gamble et al. [15]. The SAP (version 2 July 2022) was written in conjunction with the NEON protocol (version 7 18/05/21). The revision to version 1 was done blind to any treatment information or emerging results. The revision included the reporting of dropouts, withdrawals and engagement by gender and ethnicity and the subgroup analysis with gender for the primary outcome [16].

## Methods and design

### Study objectives

The primary objective is to evaluate the effectiveness of the NEON Intervention in improving quality of life at 1 year (mean score of the MANSA [11]) compared to usual care in people with psychosis (NEON Trial) and people with non-psychosis mental health problems (NEON-O Trial).

Secondary objectives include: evaluating effectiveness in improving hope (total item score of the Herth Hope Index, [17]), empowerment (total item score of the Mental Health Confidence Scale, [18]), meaning in life (mean item score for presence and search subscales of Meaning of Life questionnaire, [19]), reducing symptomatology (total item score of the CORE-10 questionnaire, [20]) and a generic preference-based health status measure, the mean index score of the 5-item EQ5D-5L [21, 22] at 1-year follow-up; to describe how the intervention is used and experienced; and to determine whether the effectiveness of the NEON intervention varies according to prior health-service usage or gender for the primary outcome of mean score on the MANSA.

### Trial design

The protocol for the NEON trials has been published [23, 24]. In brief, both trials are two-arm parallel group randomised superiority trials to determine whether the NEON Intervention improves the quality of life at 1 year on the MANSA questionnaire, compared to a usual care control arm.

No formal interim analysis comparing any outcome data between the intervention and control group is planned. The final analysis will take place once all the follow-up data have been collected and the database has been locked.

### Inclusion and exclusion criteria

The NEON Trial is conducted in a population that has experienced psychosis in the last 5 years. The NEON-O Trial is conducted in a population that has experience of non-psychosis mental health problems in the last 5 years. Eligibility criteria are described in Table 1.

**Table 1:** Inclusion and exclusion criteria for NEON and NEON-O Trials

	**NEON**	**NEON-O**
**Inclusion criteria**	1. Experience of psychosis in the last 5 years	1. Experience of mental health problem other than psychosis in the last 5 years
	2. Experience of mental health-related distress in the previous 6 months	2. Experience of mental health-related distress in the previous 6 months
	3. Resident in England	3. Resident in England
	4. Aged 18 or above	4. Aged 18 or above
	5. Capable of accessing or being supported to access the internet, either on a personal computer, mobile device or at a community venue	5. Capable of accessing or being supported to access the internet, either on a personal computer, mobile device or at a community venue
	6. Able to understand written and spoken English	6. Able to understand written and spoken English
	7. Capable of providing online informed consent	7. Capable of providing online informed consent
**Exclusion criteria**	None	1. Eligible for NEON Trial

### Intervention

Participants in the control arm received no changes to any treatment they may be receiving.

Participants in the intervention arm will receive usual care plus access to the NEON Intervention. The NEON Intervention is an online, password-controlled interface that presents mental health recovery narratives to participants. The NEON Intervention can be accessed through a web-browser on a computer or mobile phone. Participants in the intervention group can access a recovery narrative via six routes: (i) they can request the automated recommendation of a narrative (personal profile information and the feedback they and others provide about each narrative will inform future recommendations), (ii) they can choose to see a randomly selected narrative, (iii) they can self-select a narrative after browsing narratives grouped by categories, (iv) they can request a previously bookmarked narrative, (v) they can access a narrative via a monthly email with specific narrative recommendations, or (vi) they can access a narrative that has been rated as hope-inspiring. Details of how the model trained by the machine-learning algorithm developed through the trial (machine learning analysis) will be published separately.

After 52 weeks, both treatment groups receive immediate/continued access to the NEON intervention.

### Blinding

Participants were not blinded to their treatment allocation. The statisticians and Chief Investigator were blind to treatment allocation. Participants entered demographic and outcome data directly into the web-based data collection tool; interaction with trial researchers was minimal. The trial team provided technical support where required and researchers followed up with participants via phone or email to collect the 52-week primary outcome data where this had not been entered into the data collection tool. The trial statistician was unblinded to treatment allocation after all data had been collected and the statistical analysis plan had been formally finalised.

### Randomisation

Participants were randomised centrally in a 1:1 ratio by permuted blocks of randomly varying lengths between 2 and 6. No stratification of participants on any baseline covariates was conducted.

### Power and sample size

The NEON Trial is powered on mean item score for the 12 subjective items in the MANSA. The primary endpoint for the NEON Trial is a minimal clinically important difference (MCID) in the mean item score. This is defined as an average difference in MANSA scores between the two groups of 0.25 (SD = 0.9 [25]) at 1-year follow-up, this can alternatively be specified as Cohen’s d of 0.27 [25]. A total sample size of 684 (342 participants per arm) will provide 90% power to detect a minimal clinically important effect size (Cohen’s d) of 0.27 (SD = 0.9, power = 90%, significance level = 0.05), allowing for 20% attrition. This will give an analysable sample of 546 (273 participants per arm).

The NEON-O Trial started as a feasibility study. However, due to its high recruitment rate, it was extended to a definitive study. The documentation of this decision is provided in an update to the protocol [23]. A sample size was then calculated for the NEON-O Trial definitive study powered on the mean item score for MANSA, and the same MCID as for the NEON Trial. The standard deviation of MANSA for the study population has been estimated from baseline data provided by the first 350 participants enrolling in the trial, who were recruited whilst this was still considered a feasibility study. A total sample size of 994 (497 participants per arm) will provide 90% power to detect a minimal clinically important effect size (Cohen’s d) of 0.27 (SD = 0.94, power = 90%, *p* = 0.05), allowing for 40% attrition. This will give an analysable sample of 596 (298 participants per arm). The attrition rate was estimated from completion rates for week 1 and week 12 data, where available for the first 350 participants.

### Outcomes

Table 2 summarises the primary and secondary outcomes, their collection timepoints and visit windows. Outcomes were considered representative of the scheduled timepoint if they were collected within + 7 days for the week 1 timepoint, within + 31 days for the week 12 and within + 90 days for the 52-week timepoint. The 52-week visit window was to allow sufficient time to collect outcome data and was informed by process evaluation interviews, which indicated that societal change due to COVID-19 has impacted on response rates for some participants such as domestic routines changing due to participants returning to office work.

**Table 2:** Outcomes measures and collection timepoints

Domain	Measure	Number of items	Endpoint	Improvement	Collection timepoint (visit window)
**Primary outcome**					
Quality of life	Manchester short assessment of quality of life [11]	12	Mean item score Range 1–7	Higher scores	0 baseline 1 week (+ 7 days) 12 weeks (+ 31 days) 52 weeks (+ 90 days)
**Secondary outcomes**					
Symptomatology	CORE-10 [20]	10	Total item score Range 0–40	Lower scores	0 baseline 52 weeks (+ 90 days)
Hope	Herth Hope Index [17]	12	Total item score Range 4–48	Higher scores	0 baseline 52 weeks (+ 90 days)
Empowerment	Mental Health Confidence Scale [18]	16	Total item score Range 16–96	Higher scores	0 baseline 52 weeks (+ 90 days)
Meaning in life	Meaning in Life Questionnaire [19]	10	Mean item score for presence and search subscales Range 1–7	Higher scores	0 baseline 52 weeks (+ 90 days)
**Health Economic measures**					
Health-related quality of life	EQ-5D-5L [21, 22]	5	Mean index score Range − 0.285–1.00 Mean domain scores (1–5)	Higher scores	0 baseline 52 weeks (+ 90 days)
Service use	Client Service Receipt Inventory (abridged) [26]	10	Mean item score, overall cost per user	Lower scores	0 baseline 52 weeks (+ 90 days)

## Statistical principles

All data analysis will be carried out using the statistical software R. [27].

Normally distributed data will be summarised by mean (standard deviation), non-normally distributed data will be presented as median (interquartile range) and categorical variables presented as n (%).

Hypothesis tests will be two-sided and estimated treatment effects will be accompanied by a 95% confidence interval. For all analyses, a significance level of 5% will be used.

### Analysis population

The enrolment of study participants was conducted completely online. This gave potential for participants to register for the trial multiple times using alternative email addresses, be this for access to treatment, financial gain (receiving multiple payment vouchers) or by human error. We refer to this as repeat registration. Potential repeat registrations were monitored throughout the trial using a protocol agreed with a Programme Steering Committee (PSC) and Trial Management Group (TMG). The details of the approaches and procedures for detecting and managing repeat registrations within the NEON trials will be published separately.

In the situation where repeat registrations appeared to have been made for profit then all accounts were suspended. Where cases of repeat registration for access to the intervention were made by a participant considered clinically vulnerable, then the account obtaining access to the intervention was allowed to continue, and all other related accounts were suspended.

We used a modified intention-to-treat (ITT) population, excluding all suspended accounts. The per protocol population will additionally exclude participants that have one or more of the following protocol deviations:

1. Participants in the intervention group that have been identified as having a repeat registration for access, but where the intervention group account has been retained.
2. Randomised in error participants. Participants who should not have entered the trial due to misunderstanding in completing eligibility questions, (e.g., participants in the NEON Trial who have not had psychosis in the 5-year period pre-randomisation).
3. Participants in the control group who briefly obtained access to the NEON intervention due to a fault in the NEON web-application which affected its operation at the beginning of trial.
4. Participants that completed the 52-week outcome data later than 90 days after the scheduled visit

### Accounting for missing data

As the data collection was performed online, validation measures built into the system mean that incomplete questionnaires could not be submitted, so items within a questionnaire cannot be missing.

Missing data will be accounted for by multiple imputation (MI) under the assumptions and conditions that follow.

#### Multiple imputation assumptions

The missing data mechanism will be assumed to be missing at random (MAR), provided some strong predictors of missingness are present for each outcome and no prior knowledge exists that would suggest a violation of this assumption i.e., that observations are missing not at random (MNAR). The baseline data available is summarised in the statistical analysis section that follows.

If we are unable to identify appropriate predictors, or missingness is less than 5% for the primary outcome, an available case analysis will be carried out [28].

If attrition between control and intervention arms differs by more than 5% controlled MI will be explored for an analysis under missing not at random (MNAR).

#### Description of missing data

The number (percentage) of missing demographic data and clinical outcomes at baseline and follow-up time points will be summarised, and any prior, trial-based, knowledge around reasons for missing data described and discussed.

#### Predictors of missingness

A multivariate logistic regression, including all demographic and clinical variables at baseline will be used with missingness indicator as an outcome for each outcome. Any variables which are statistically significant at 5% will indicate a strong predictor for missing data in that outcome.

#### Multiple imputation

The process of multiple imputation by chained equations will be performed once to impute all missing baseline and clinical outcomes using the MI package in R. The number of datasets generated (at least 5) will reflect the percentage of missing data present and chains will be assessed for convergence to choose a suitable burn-in period to remove before official convergence tests on multiple chains.

The imputation model will include variables identified to be predictors of the missing data mechanism; auxiliary variables correlated with the outcome, and all variables contained in the substantive model. For continuous outcomes, truncated regression will be used to ensure imputed data do not extend beyond the range of the questionnaire. The imputation model will account for the longitudinal nature of the outcomes by selecting the nearest (in time) available data of the same outcome as a covariate.

Individual analyses on each imputed dataset will be combined using Rubin’s rules.

#### Assumption checking

Multiple imputation (MI) will be undertaken if we have strong predictors of missingness and an imputation model that is predicting plausible values. Diagnostic checks will be performed on the imputation model to compare the distributions of imputed values with the non-missing values. These checks may be done graphically or via summary statistics.

## Statistical analysis

### Participant flow

**Fig. 1:**
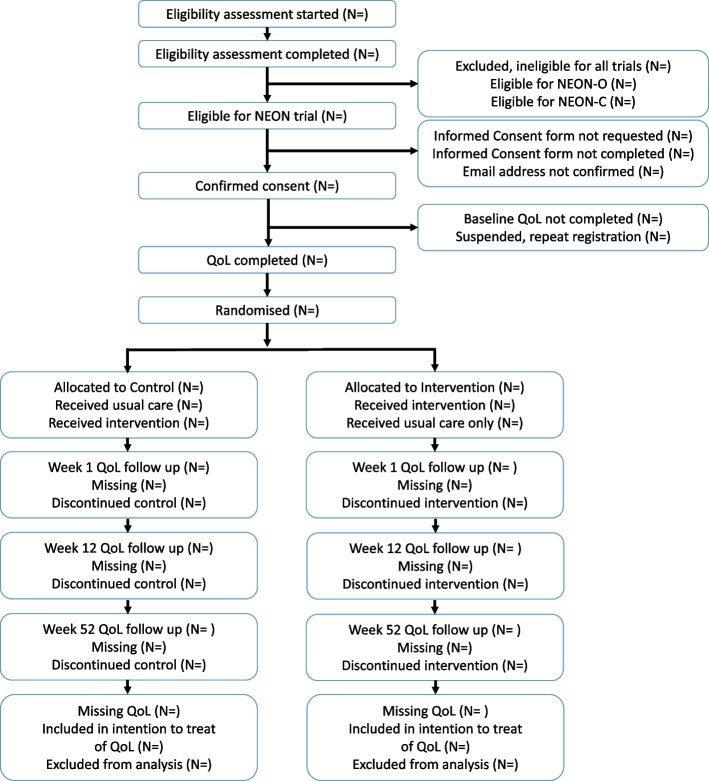
CONSORT flow diagram

Participant flow through the trial will be summarised by a CONSORT flow diagram at the end of the trial (Fig. 1). [29] Data on suspended accounts due to repeat registration, withdrawals from intervention and/or follow-up (with timing and reasons, where known) and loss to follow-up will also be presented separately by gender and ethnicity [16].

### Baseline data

Demographic information including age, gender, ethnicity, geographical region in England, occupation, living arrangements, highest qualification, recovery status, service use, and main mental health problem in the last month will be summarised by treatment group, with no formal hypothesis testing for group differences. Clinical outcomes are described in Table 2 and additional summaries will be provided for the Education and Living Alone items of the MANSA which are not included in the QoL calculation.

### Recruitment

The recruitment route used (participant indicates a promotion in primary care, a promotion in secondary care, other mechanisms, or clicked on a link in an online advert) and the recruiting NHS Trust will be summarised for the total number of eligible participants.

### Treatment adherence

For those in the intervention group, “received intervention” will be summarised by ever having accessed a narrative in the NEON collection. For those in the control, “received control” will be calculated by participants never having accessed any narratives in the NEON collection; this will be summarised in the CONSORT diagram.

The number of participants reporting access to recovery narratives outside of the NEON Collection is collected at week 1, week 12 and week 52. It will be reported overall and by the treatment group at each timepoint.

### Treatment engagement

For those in the intervention group, engagement will be summarised as the median (and range) number of times a participant (a) logs into the NEON Intervention, (b) receives a recovery narrative (all narratives and unique narratives), and (c) provides narrative feedback (all narratives and unique narratives), up to the collection of the primary outcome at 52 weeks. These figures will also be presented separately by gender and ethnicity.

### Protocol deviations

The number of participants deviating from the protocol according to each criteria defined above, and the number of participants with at least one deviation, will be summarised by treatment group.

### Primary and secondary analysis

The primary outcome is the mean score of all subjective MANSA items at 52 weeks. Descriptive statistics will be presented by the treatment group for weeks 1, 12 and 52 data. The primary analysis will be a linear regression model of the outcome at 52 weeks adjusting for baseline score, with hypothesis testing on the regression coefficient for treatment and missing data accounted for by multiple imputation. The results will be presented as adjusted difference in score at 1-year follow-up with associated 95% confidence intervals.

The secondary outcomes are total scores at 52 weeks of the CORE-10, Herth Hope index, Mental Health Confidence Scale, the mean item scores for the two sub-scales for the meaning in life questionnaire and the total index score for EQ-5D-5L. The National Institute for Health and Care Excellence (NICE) recommends EQ-5D-3L, not EQ-5D-5L, in its reference case [30, 31]. NICE supports prospective clinical studies using EQ-5D-5L to collect data on health status and recommends a 5L to 3L crosswalk algorithm for reference-case analyses, as opposed to the English 5L value set, to estimate QALYs [32]. Therefore, in this study, two sets of EQ5D-5L values are derived using both the EQ-valuation technology (VT) method with the England reference table and the crosswalk (CW) method using the UK reference table. Descriptive statistics will be presented by the treatment group.

The analysis of each secondary outcome will be a linear regression model of the outcome at 52 weeks adjusting for baseline score, with hypothesis testing on the regression coefficient for treatment. The results will be presented as adjusted difference in score at 1-year follow-up with associated 95% confidence intervals.

To check the suitability of the linear regression models diagnostic plots (normal Q-Q plot, residuals versus fitted values, and residuals versus leverage) will be produced to assess the normality of residuals, independence of errors, linear relationship between the outcome and predictors, homogeneity of variance of residuals, and to check for influential data points. If departures from these assumptions are seen alternative methods such as transformations, generalised least squares estimation, or non-parametric methods will be explored, dependent on the type, and extent of departure.

### Subgroup analyses

The trials have not been powered to detect subgroup effects; hence these should be considered exploratory. To explore any differential treatment effects the primary analysis will be repeated twice, first to include an interaction term between treatment and service user type at baseline, and second to include an interaction term between treatment and gender (male, female, other). Service user type will be defined as specialist service use or no specialist service use, where specialist service use is defined as having ever (including currently) used specialist mental healthcare services, e.g. a community mental health team or mental health in-patient ward.

The presence of an interaction will be tested using a likelihood ratio test comparing the subgroup analysis model, including the interaction term, and the primary analysis model, not including the interaction term. The test result will be considered significant at the 5% level. Within each category of service user type, we will report summary statistics of the outcome by treatment arm, a treatment effect and 95% confidence interval.

### Sensitivity analyses

As a sensitivity analysis around the MI imputation model, we will carry out a complete case analysis of the primary outcome with the significant predictors for missingness added as covariates. The imputation may be more susceptible to the assumptions made at higher values of missingness. In this case, we will conduct a sensitivity analysis around the MAR assumption using the approach described by Carpenter [33].

A per protocol analysis will be carried out and compared to the ITT analysis for all protocol deviations and also for each protocol deviation individually for the primary outcome only.

To investigate the effect of nationally imposed restricted movement and social contact (referred to as lockdowns) during the COVID-19 pandemic, we will compare baseline clinical data and the cross-walked EQ-5D-3L (calculated from the EQ-5D-5L following NICE guidelines) collected during times of national lockdown to those collected outside of lockdown and also summarise the number of participants completing baseline data and average baseline MANSA by the week of the trial, highlighting periods of national lockdown.

We will conduct a linear mixed model for repeated measures for the primary outcome with the lockdown (yes/no) included as a covariate; baseline will be included as a time point (but the treatment effect only estimated for the follow-up timepoints). The analysis will be repeated for the cross-walked EQ-5D-3L.

National lockdown dates are defined as follows: lockdown 1: 23 March 2020 to restrictions easing 15th June 2020; Lockdown 2: 5th November 2020 to 2nd December 2020; and Lockdown 3: 6th Jan 2021 to restrictions easing 8th March 2021.

### Safety data

The number of serious adverse events, their relationship to the intervention, and whether the event is expected or not will be summarised by the treatment arm.

### Further analyses

Further analyses of a more exploratory nature will not be bound by this analysis plan but will broadly follow the principles laid down in it. Any post hoc analysis, such as those requested by journal reviewers, will be clearly labelled as such in the final trial reporting.

## Discussion

As both trials were conducted during the pandemic, usual care during the pandemic may not have reflected usual care pre-pandemic. For example, for the participants using mental health services, there was greatly restricted access to services, much of the support moved to online rather than in-person meetings, and the experience of in-patient admission processes changed to reduce the spread of COVID-19 [34]. Statistical consequences include secular effects such as participants entering the study at different time points having different access to treatment as usual and different likelihood of COVID-related mental health issues [35]. This leads to the potential for sampling bias [36] and may influence the proposed treatment effect.

The collection of additional data to determine the impact of the pandemic on the data collected for the NEON trials was not possible. This could mean that important predictors of missingness are not available for our imputation model, and this may impact upon whether a suitable imputation model can be built for this data.

In the NEON trials, consent was conducted completely online and therefore a participant’s identity was not independently validated; only a valid email address was required for consent. This approach is in keeping with the HRA and MHRA joint statement on seeking and documenting electronic consent [37]. This meant that participants could use the NEON web-application anonymously. This led to the potential for re-registration into the trials either by human error, to access the intervention or for the financial incentive (a £20 voucher was offered to participants on completion of measures at each timepoint). Repeat registrations undermine the trial randomisation, is a misuse of public money, and the possibility of participants providing false information to gain access into the trial could undermine the validity of the data. Strategies that implement a form of offline identity verification could be implemented to alleviate these issues. We did not implement this in NEON as the ability to use the system anonymously is particularly important to our trial population since people with psychosis may be vulnerable to concerns about online data usage and may also fear the stigma associated with mental health problems [38].

A potential issue with online trials is that the researcher has little control in how the intervention is used, and if a large proportion of participants do not use the intervention, then the treatment effect is diluted. The NEON intervention is designed for participants to use as little or as frequently as they wish; there is no expected pattern of usage. It is not known whether receiving more narratives translates to effective engagement or whether receiving fewer but more targeted narratives could result in a positive change in outcome. The NEON Intervention automatically records detailed information regarding intervention use, such as each start/end time using the intervention and the number of narratives accessed. The process evaluation will provide an understanding of patterns of receiving recovery narratives and explore their relationship with outcomes. For this analysis, we take a pragmatic approach, and the treatment effect is calculated regardless of intervention use.

Without the personal contact with the study team, online trials can face problems with retention of participants and with increased loss to follow-up comes a reduced in power. A higher estimate for the proportion loss to follow-up was used in the sample size calculation for the NEON-O trial based on the data seen in the feasibility study. Reimbursement for participation at the primary endpoint was then introduced for NEON-O as a definitive trial to reduce loss to follow-up. Researchers followed up participants via phone or email to collect the 52-week primary outcome data where this had not been entered into the data collection tool to reduce missing primary outcome data. The potential for differential follow-up between treatment arms will be accounted for using controlled multiple imputation if there is a difference greater than 5%.

Most data collected in the NEON trials was done so directly via the NEON web-application. This provided advantages in that the data collection tool did not allow individual items within the questionnaire to be missing, thus reducing this type of missing data problem. The downside to the online platform, and minimal contact with the research team, it is not possible to query or resolve any data issues that may present themselves at the point of data analysis, or earlier. Alternative procedures to make assessments of the data quality can be made by taking advantage of the data automatically logged by the system. For example, the time spent by a user on a single questionnaire could indicate whether thought and time have been applied to the completion of the data or whether it has been completed overly quickly.

The UK government response to the COVID-19 pandemic was a national lockdown, restricting movement and social interactions. These were later followed by local lockdowns, further national lockdowns and ongoing social restrictions on movement and interactions and quarantining of individuals with symptoms or exposure. The impact of lockdowns and quarantine may have adversely affected the quality of life of participants and may have affected access to normal care for all participants. These effects may be different throughout the duration of the trial. We do not know the level of impact the COVID pandemic will have had upon access to the NEON intervention; participants may not be able to access the internet from home such as people dependent upon public internet access in, for example, public libraries that may have closed during lockdown. Engagement with the intervention could have been improved with people spending more time at home, but conversely, those experiencing anxiety around their health, experiencing health issues related to COVID or government restrictions, and financial worries may have lacked the capacity to fully engage with the intervention and complete data at required visits. Those who are at higher risk for worse COVID symptoms may be affected differently.

We have included sensitivity analyses to gauge whether there has been any impact on the data collected in times of intense lockdown and restricted movement compared to less restricted times. As we are unable to determine what data has been affected by pandemic-related intercurrent events, all participant data is included in the analysis which gives us an estimand of the treatment effect in a world including a pandemic, which may inform future trials, given that usual care is no longer in the same form post-pandemic.

## Data Availability

Anonymous and pseudonymous elements of the datasets used and/or analysed during the study will be available on request from the study team before the NEON study ends, although requests may be refused whilst research publications are being generated. After the NEON study ends, anonymous and pseudonymous research data will be available from the study sponsor on reasonable request until the end of the retention period, but the request may be refused if NEON study investigators are still generating research publications from this data. After the retention period, availability through the study sponsor or Chief Investigator may be provided at their discretion. Contact the study sponsor through Research@nottshc.nhs.uk.

## Abbreviations

CSRI: Client Service Receipt Inventory
ITT: Intention-to-treat
IQR: Interquartile range
MANSA QoL: Manchester Short Assessment of Quality of Life
MAR: Missing at random
MCID: Minimal clinically important difference
MI: Multiple imputation
MNAR: Missing not at random
NEON: Narrative Experiences Online
NHS: National Health Service
PSC: Programme Steering Committee
RCT: Randomised controlled trial
SAE: Serious adverse event
SAP: Statistical analysis plan
SD: Standard deviation
TMG: Trial Management Group
